# Promoting Circular Economy by Leveraging Annatto Byproducts from *Bixa orellana* L. into Sustainable Antioxidant Food Packaging

**DOI:** 10.3390/foods14040704

**Published:** 2025-02-19

**Authors:** Vanilda Aparecida Soares de Arruda-Peixoto, Paula Vera Estacho, Magdalena Wrona, Paulo Roberto Nogueira Carvalho, Roseli Aparecida Ferrari, Cristina Nerin, Elena Canellas

**Affiliations:** 1 Food Technology Institute, Av. Brasil. 2280, Campinas 13070-178, SP, Brazil; carvalho@ital.sp.gov.br (P.R.N.C.); roseliferrari@ital.sp.gov.br (R.A.F.); 2 Department of Analytical Chemistry, Aragon Institute of Engineering Research I3A, EINA—University of Zaragoza, Torres Quevedo Building, María de Luna 3, 50015 Zaragoza, Spain; pvera@unizar.es (P.V.E.); cnerin@unizar.es (C.N.); 3 Institute of Bio- and Geosciences, 2, Forschungszentrum Jülich GmbH, 52428 Jülich, Germany

**Keywords:** *Bixa orellana* L., annatto, bioactive compounds, antioxidant packaging, HS-SPME-GC-MS, food packaging, UPLC-MS/QTOF, circular economy

## Abstract

Annatto (*Bixa orellana* L.) is cultivated primarily for the extraction of bixin, a natural dye with substantial industrial importance, resulting in the generation of large quantities of residues that remain underutilized. This study provides the first in-depth characterization of annatto byproducts derived through molecular distillation, highlighting their untapped potential for sustainable innovation. Employing state-of-the-art techniques—HS-SPME-GC-MS for volatile compounds and UPLC-MS/QTOF for non-volatile ones—the research identified a remarkable array of bioactive constituents. Over thirty pharmacologically significant compounds were unveiled, many appearing for the first time in annatto byproducts. Notable discoveries include diterpenoid alcohols, oleamide, δ-tocotrienol, n-alkanes, fatty acid methyl esters, and springene among the volatiles. Among the non-volatiles, groundbreaking identifications such as dihydroactinidiolide, dihydrochalcone, 3-phenyl propiofenone, novel tetracosan amides, halisphingosine A, kauranetriols, and phytoene derivatives redefine the chemical profile of this residue. Further amplifying the value of these findings, the study successfully transformed these byproducts into innovative antioxidant packaging materials, demonstrating their high potential for food preservation and sustainable applications. The packaging films, developed from samples devoid of vegetable oil, exhibited robust antioxidant properties, offering a compelling solution to extend shelf life and reduce spoilage. This work underscores the importance of revalorizing agricultural residues like annatto byproducts, turning waste into high-value resources that align with the principles of the circular economy.

## 1. Introduction

The annatto plant (*Bixa orellana* L.), a member of the *Bixaceae* family, is native to tropical America and has been widely distributed from the Guianas to Brazil. Traditionally utilized by indigenous peoples for dye extraction, annatto seeds are now commercially exploited for their pigment bixin, with global production estimated at approximately 14,500–17,000 tons annually. Bixin is widely used in the food, pharmaceutical, and cosmetic industries, accounting for around 70% of the global market for natural colorants, highlighting its significant industrial and economic value. The pigment is concentrated in the pericarp or aril, which constitutes approximately 5–10% of the seed’s total weight, making it a key economic component. Bixin content can vary between 1% and 6%, depending on the cultivar, climatic conditions, and regional soil characteristics, but is critical for commercial viability, with a minimum threshold of 3% bixin necessary for profitable production [1]. While bixin extraction remains the primary focus of annatto seed processing, the potential of other applications has been recently studied. For instance, its possible antimicrobial activity has been investigated by Steiner et al. [2]. Annatto seeds, beyond their primary pigment content, are rich in bioactive compounds such as lipids and antioxidants, which offer significant potential for innovative applications like active packaging materials with antioxidant and antimicrobial properties. These materials can extend the shelf life of food products, improve packaging sustainability, and reduce dependence on complex, non-recyclable plastics [3].

Additionally, the circular economy represents a transformative paradigm designed to address the limitations of the traditional linear “take–make–dispose” model. It emphasizes the responsible and efficient use of resources, coupled with the valorization of byproducts rich in valuable compounds. Both practitioners [4] and researchers highlight the circular economy’s dual objectives of reducing environmental impacts and enhancing human well-being. However, prevailing interpretations of the circular economy often adopt a narrow focus, primarily centered on optimizing waste management practices. This perspective tends to prioritize activities such as “enhanced” recycling, recovery, and reuse, which, while valuable, may not fully achieve the circular economy’s broader goals. Nevertheless, revalorizing industrial residues as byproducts is essential for fostering a circular economy, as it promotes resource efficiency, minimizes waste, and supports the sustainable reuse of materials across industries. The concept of a circular economy, which promotes the reuse of byproducts and waste reduction, is gaining increasing relevance within the agri-food and related industries.

The byproducts of the annatto dye extraction, often undervalued, may include valuable compounds. The valorization of these secondary compounds aligns with the growing global emphasis on sustainability and resource efficiency, offering new avenues for minimizing waste and maximizing the economic utility of annatto seed processing residues [5,6]. The integration of annatto seed byproducts, such as the unsaponifiable fractions of annatto oil, into sustainable packaging solutions highlights the broader importance of the circular economy. Transforming these byproducts into valuable biodegradable films not only preserves food quality but also contributes to the demand for environmentally sustainable materials, reducing environmental impacts and generating new economic value [7,8]. This approach plays a critical role in advancing bio-based packaging materials and further supports the transition toward a more circular and sustainable economy.

In this study, various types of byproducts have been characterized, for the first time. Their antioxidant properties have been studied, and active plastic films containing these byproducts have been developed. The characterization involved a detailed analysis of the chemical composition of the byproducts, identifying key bioactive compounds with potential applications in sustainable materials. The antioxidant properties were evaluated through in situ gas-phase hydroxyl radical generation assays [9] to determine their efficacy in inhibiting oxidative processes, which is critical for extending the shelf life of packaged products. This study will explore, for the first time, the potential of integrating annatto agricultural byproducts into sustainable packaging solutions, presenting a dual advantage of waste valorization and the innovation of functional materials. This approach marks a substantial advancement in the development of eco-friendly packaging technologies, with far-reaching implications for reducing environmental impact while enhancing food safety and quality.

## 2. Materials and Methods

### 2.1. Materials

#### 2.1.1. Industrial Annatto Residue

**Figure 1:**
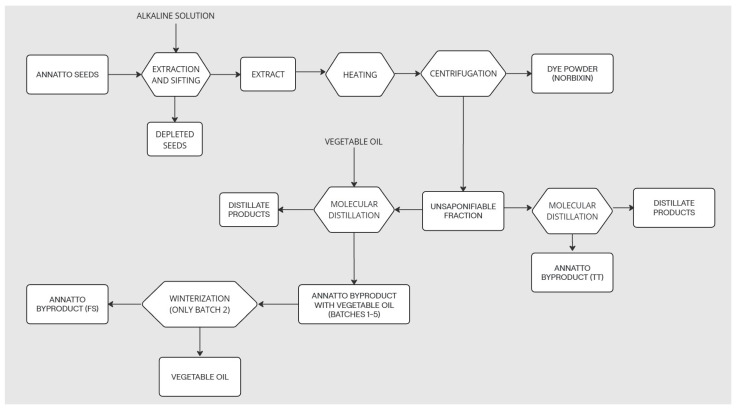
Production process flowchart.

Five samples of annatto byproducts were provided by the company New Max Industrial LTDA, an annatto dye industry located in the city of Americana, SP, Brazil. Annatto seeds undergo a saponification process with an alkaline solution to obtain annatto dye (norbixin). During the extraction process, in addition to the dye, unsaponifiable material from annatto seeds is obtained. This unsaponifiable material is subjected to molecular distillation to separate terpenes and herbal medicines such as tocotrienols. The molecular distillation process occurs in two distinct routes. In one of them, vegetable oil is added as a process vehicle. Five samples of waste containing vegetable oil were obtained at the end of this process and were identified as Batches 1–5. A sample of annatto residue was obtained after molecular distillation without the addition of vegetable oil and identified as TT. Another sample was prepared in the ITAL laboratory from the byproduct sample of Batch 2 of annatto through the winterization process and identified as FS (solid fraction). The extraction procedure is shown in the Figure 1.

#### 2.1.2. Reagents

The reagents used in the extraction procedures and other analytical methods were of analytical or chromatographic grade, as required. Ultrapure water was prepared using the Milli-Q Direct purification system (Millipore, Bedford, MA, USA). Standard C7 to C40 alkanes (49452-U), tridecane (CAS 629-50-5), eicosane (CAS 112-95-8), 9-octadecenoic (Z); methyl ester; 5-hepten-2-ona,6-methyl; *p*-xylene; *o*-xylene; 5,9-Undecadien-2-one, 6,10-dimethyl-, (E); 2,6,10-Dodecatrienal, 3,7,11-trimethyl-; and cis-beta-Farnesene were supplied by Sigma-Aldrich (Madrid, Spain).

### 2.2. Methods

#### 2.2.1. Sample Preparation

- Isolation of precipitable compounds from the industrial annatto residue

The winterization method according to [10] was used with modifications. About 1 g of the lipids extracted from the industrial annatto residue (in hexane) were weighed into centrifuge tubes and dissolved in an acetone: hexane (85:15) solution in a proportion of 1:20. The solutions were maintained under refrigeration at −15 °C for 7 days to precipitate the compounds present in the annatto byproduct, centrifuged at 9000 rpm/min for 15 min at −10 °C, the supernatant discarded, and the tubes containing the precipitate maintained in an incubator at 55 °C for 2 h before weighing the solid fraction.

- Hexane extraction

A total of 50 mg of each sample was diluted in 10 mL hexane and the solution maintained in an ultrasonic bath (frequency 40 KHz) for 60 min. The solutions were then filtered through a 0.22 µm PTFE membrane before injecting into the chromatograph. A blank solution was prepared together with the samples and the samples analyzed in triplicate.

- Methanol and dichloromethane extraction

A 250 mg sample was diluted in methanol and dichloromethane (3 mL). The solutions were shaken for 3 min, maintained in an ultrasound bath (frequency 40 KHz) for 60 min, filtered through a 0.45 µm PTFE membrane, and diluted (1:100) before injection. Blank solutions were prepared together with the samples and three replicates of each sample analyzed.

- Headspace-solid phase microextraction (HS-SPME)

A total of 2 g of each sample was weighed directly into 20 mL empty flasks for analysis. Blank solutions were prepared together with the samples and two replicates of each sample analyzed.

#### 2.2.2. HS-SPME-GC-MS and GC-MS

For the Headspace-solid phase microextraction—Gas Chromatography—Mass Spectrometry (HS-SPME-GC-MS) method, the HS-SPME extraction was carried out using a DVD/CAR/PDMS fiber at 80 °C for 15 min.

An Agilent Technologies (7820A) gas chromatograph was used, coupled to an Agilent Technologies serial mass detector (MSN 5977B) (Madrid, Spain). An Agilent Technologies (Madrid, Spain) model HP-5MS capillary column (30 m × 0.25 µm × 250 µm) was used for the chromatographic separation, the injector temperature was 250 °C, injection was in the splitless mode, and the helium flow rate was 1.0 mL/min. The oven temperature program was as follows: Initial 50 °C (5 min), temperature ramp of 10 °C/min to 300 °C, maintaining this temperature for 5 min, with acquisition in the SCAN mode (50–450 *m*/*z*).

Direct injection was used for the Gas Chromatography—Mass Spectrometry (GC-MS) analysis, injecting a sample volume of 1 µL, under the same analytical conditions described above. Solvent delay was 5 min.

The identification of the compounds obtained by GC-MS and HS-SPME-GC-MS was obtained by the comparison of the spectra with the NIST MS Search 2.4 library spectra results in a match greater than 85%. Identifications were confirmed with pure standards when they were commercially available.

#### 2.2.3. Analysis of the Antioxidant Activity of the Samples

The antioxidant activity of all the samples was measured using a previously published method [9]. The configuration consisted of a Bio-Rad peristaltic pump (Hercules, CA, USA) adjusted to 0.8 mL/min and total air flow adjusted to 3.76 L/min. A photoreactor was used to generate OH• radicals from a 1.66% (*v*/*v*) hydrogen peroxide solution. The photoreactor consisted of a 300 × 30 mm cylindrical quartz tube irradiated by ultraviolet light generated by eight UV lamps, and 250 × 15 mm fluorescent Philips tubes (TL 8 W/08 F8T5/BLB Hg, Eindhoven, Netherlands) were placed axially round the quartz tube.

The samples were prepared according to a previously published method [9]. A 10 µL aliquot of each sample was added to a Pasteur pipette containing 0.3 g of glass wool and the pipette connected to the OH• radical generator. Drechsler-type gas purifying bottles containing 50 g of a 2 µg/g sodium salicylate solution were used to collect the combustion gases, analyzing the samples in triplicate. Samples with antioxidant potential were compared to the blank, which consisted of the same laminates that contained adhesive without the annatto extract, and oxidation of the samples was carried out for 24 h.

The solutions were injected into the HPLC chromatograph coupled to a fluorescence detector using the following chromatographic conditions: column—AtlantisTM C18 (100 mm × 4.6 mm i.d. × 5 µm); mobile phase—acetate buffer (35 mmol/L; pH = 5.9): methanol (9:1); column temperature—25 °C; sample temperature—25 °C; flow rate—1 mL/min; mode—isocratic; injection volume—10 µL; λ ex = 324 nm; λ em = 448 nm. The percent hydroxylation was obtained as the result, calculated using a rule of three, where 100% is the area of the 2,5-DHB peak in the blank, and x% is the area of the 2,5-DHB peak in a sample with antioxidant activity.

#### 2.2.4. Preparation of an Active Package with an Active Coating Layer

The extract concentration (annatto residue sample) was 5% (weight/weight) in a water-based adhesive (AXILAT L4805 from Samtack SL). The sample aliquot was added to the glue in a falcon tube, homogenized with a spatula, heated at 60 °C for 4 h to dissolve better and left to rest for 24 h. The active adhesive was then spread on a PRT CHEM sheet using a K Control Coater (RK Print Coat Instruments, Litlington, UK) using the nº 2 bar and a velocity of 5 (m/min).

The samples were prepared, and the active films evaluated as follows: 1 dm^2^ of each sample was placed in an LDPE/PA bag (13 × 13 cm) with a micropipette tip inlet and outlet and the bags placed in separators [9,11]. The bag inlets were subsequently connected to an OH• generator and bottles containing sodium salicylate. The measurements were taken against blanks (1 dm^2^ of pure coated PET without antioxidant).

#### 2.2.5. UPLC-MS/QTOF Analysis

Samples extracted with methanol were analyzed by ultra-performance liquid chromatography coupled with a hybrid quadrupole orthogonal time-of-flight mass spectrometer (UPLC-MS/QTOF). The chromatography was carried out using an Acquity^TM^ system coupled to a Xevo G2 QTOF detector (Waters, Milford, MA, USA). The detector consisted of an API (atmospheric pressure ionization) power supply with an electrospray interface (ESI), coupled to a Xevo G2 mass spectrometer with one hexapole, one quadrupole, a collision cell, and a time-of-flight analyzer (QTOF). A Waters Acquity UPLC BEH C18 column with a particle size of 17 µm (2.1 mm × 100 mm) (Milford, MA, USA) was used for the chromatographic separation. The solvents of the mobile phase were water (phase A) and methanol (phase B), with or without 0.1% formic acid for positive and negative ionization, respectively. The flow rate through the column was 0.3 mL/min and the column temperature 35 °C. The gradient used was 98%—2% phase A—phase B, initially terminating in 100% phase B after 15 min. The sample volume injected was 5 µL. The instrumental parameters were as follows: the electrospray probe was used in the modes positive (ESI+) and negative (ESI-) and in the sensitivity activity mode. The mass range considered was from 10 to 1000 Da. The corona voltage was 2.5 kV for (ESI+) and 0.5 kV for (ESI-) two cones. The voltages used for each injection were 30 and 75 V in order to detect a larger number of compounds. The source temperature was 150 °C, the desolvation gas temperature was 450 °C, and the outflow desolvation gas temperature 650 Lh-1.

The MS^E^ mode was selected for acquisition, which alternates between two functions: function 1, acquisition at low energy to obtain exact spectra of mass precursor ions and function 2, acquisition at high energy levels to obtain ion fragments of exact mass. The collision ramp energy was from 15 to 40 V.

MassLynx software v.4.1 (Waters, Milford, MA, USA) was used to analyze the samples, and CromaLynx (Waters, Milford, MA, USA) to deconvolve the spectra.

Qualitative analysis of the compounds was carried out first to determine the element composition of the spectrum obtained in the first function chromatogram, and second to determine the molecule of the mass fragment coinciding with the spectrum obtained in the second function chromatogram. The transformation function (TOF) was applied to spectra derived from the chromatograms of the second function (only for the ESI+ mode). This tool represents the isotopic masses and realigns them to a single state-of-charge mass axis. The databases ChemSpider^®^ and SciFinder^®^ were used to determine the chemical structures of the molecules corresponding to their chemical formulae, sorted by the number of references. Up to ten of the most referenced molecules were chosen from each databank. All the qualitative results were compared with data published in the literature

## 3. Results and Discussion

### 3.1. GC-MS

To obtain a comprehensive and detailed analysis of the sample’s chemical composition, we implemented different extraction techniques using both solvent-based methods and solid-phase microextraction (SPME). The choice of extraction methods was driven by the need to capture a broad range of compounds with varying chemical properties, ensuring that both volatile and non-volatile constituents were effectively recovered. Solvent extractions were carried out using different solvents to selectively isolate compounds based on their polarity. This approach allowed for the separation and identification of a diverse array of chemical components, including non-polar hydrocarbons, moderately polar fatty acids, and more polar bioactive molecules. Each solvent was selected to optimize the extraction of specific groups of compounds, thus improving the overall efficiency and sensitivity of the analytical process

In contrast, SPME was employed as a solvent-free technique to specifically target volatile and semi-volatile compounds. This method is particularly advantageous because it minimizes sample preparation, reduces the risk of contamination, and enhances the detection of thermally labile or highly volatile substances that might be lost during conventional solvent extraction. The use of SPME in combination with solvent extractions allowed us to obtain a more complete chemical profile of the sample, ensuring that both volatile aroma compounds and less volatile bioactive substances were effectively analyzed. By integrating these complementary extraction strategies, we aimed to enhance the accuracy and reliability of our results, providing a more holistic understanding of the sample’s chemical composition.

#### 3.1.1. Alkanes and Hexane-Soluble Compounds

Table 1 summarizes the identification of n-alkanes and hexane-soluble compounds present in industrial annatto residue samples (byproducts), as determined by GC-MS analysis. Match indicates the percentage of alignment with the NIST library.

Thirty-two compounds were identified in the search for alkanes and hexane-soluble compounds present in the samples of annatto byproduct, including hydrocarbons, fatty acids, diterpenoid alcohol, oleamide, tocotrienol, and alkanes, a total of 24 compounds with a spectral match above 85% and five alkanes with more than 28 carbons in the molecule were confirmed using the retention times of authentic standards (Table 1).

n-Alkanes, long-chain saturated aliphatic hydrocarbons, are often referred to as the “digital fingerprint” of plants, with each species exhibiting a unique profile. These stable compounds, which resist degradation better than other compounds [12], are valuable biomarkers, particularly in geological and ecological studies [13,14,15,16]. Long-chain n-alkanes (>C20) are typically derived from higher terrestrial plant waxes, while short-chain n-alkanes (<C20) are associated with fungi, algae, and bacteria. Plants predominantly produce odd-numbered n-alkanes, a pattern observed across various ecosystems [17]. However, the composition can vary significantly between and even within species [18,19]. Previous studies have identified n-alkanes with 20 to 31 carbon atoms in oils such as sunflower, corn, linseed, and soybean [20]. In this study, annatto byproducts were found to contain n-alkanes ranging from 20 to 33 carbon atoms, with the most extensive range identified in the original sample. Therefore, the distinct pattern of n-alkanes identified in annatto byproducts underscores their utility as chemical markers. This unique n-alkane profile not only provides insight into the biochemical composition of annatto but also offers potential applications in tracing the origin and processing of annatto byproducts in industrial and ecological contexts.

The most abundant compound was (E)-β-farnesene, part of the farnesene family, which consists of several isomers, including α-farnesene found in fruit skins and annatto seeds [21]. Farnesene and its derivatives, such as farnesol and farnesyl acetate, are also present in annatto essential oils [22]. This versatile compound is used in various industries, including biofuels, cosmetics (including high-value products such as coenzyme Q10), pharmaceuticals, and food production, and can be polymerized for rubber and tire manufacturing [23,24]. This information highlights the potential for annatto byproducts to be leveraged in high-value industries, promoting sustainability and increased profitability by utilizing these compounds in diverse markets.

δ-Tocotrienol was present in all the samples analyzed. It is a key compound found in annatto oil, and it is a powerful antioxidant. In most annatto samples, δ-tocotrienol was the dominant isomer, sometimes making up to 90% of the tocotrienol content, followed by γ-tocotrienol. These tocotrienols are reddish, viscous oils that are insoluble in water but dissolve in vegetable oils and non-polar solvents. Annatto is unique in having a higher proportion of tocotrienols than tocopherols, a rare trait among plants [25]. This makes annatto byproducts particularly interesting for designing antioxidant packaging materials, which could protect food products from oxidative deterioration and extend shelf life.

Both *α*- and *β-Springene*, linear diterpenes identified in all samples, are known for their wide-ranging biological activities, including antimalarial [26], antibacterial [27], anti-helminthic [28], and antineoplastic effects, particularly against breast and ovarian cancer cells [29,30]. Meanwhile, linoleic acid (9,12-Octadecadienoic acid), an omega-6 fatty acid also present in all samples, is associated with numerous cardiovascular health benefits, such as reducing heart disease risks, preventing cardiovascular and coronary conditions, and decreasing blood clots [31,32,33].

Oleamide, or 9-octadecenamide (Z), is a naturally occurring, waxy solid derived from oleic acid. It has been identified in several seed oils [34]. Oleamide is noted for its thermoregulatory and analgesic properties, and it may aid in treating mood and sleep disorder cannabinoids [35,36,37]. Research suggests that its effects resemble those of cannabinoids, and it also exhibits anti-inflammatory and antibacterial properties [38]. Additionally, oleamide may influence cardiovascular mechanisms and possess diuretic, carminative, and astringent activities [39,40].

To sum up, the presence of all these bioactive compounds makes annatto byproducts highly valuable for a variety of applications, including functional foods, pharmaceuticals, and the design of antioxidant and antimicrobial packaging materials, enhancing product preservation and health benefits.

#### 3.1.2. Methanol-Soluble Compounds

Since samples FS and TT were the non-vegetable oil diluted samples, they were also extracted with methanol to identify more polar compounds present in the samples.

Table 2 shows the compounds found in methanol extracts of industrial annatto residue from two samples (FS and TT). In sample FS, three compounds were identified with an over 98% spectral match, including the confirmed presence of 9-octadecenoic (Z) methyl ester. In sample TT, compounds identified included isomers of *springene* and 9,12-octadecadienoic acid methyl ester, with a spectral match with the NIST library above 86%.

Notably, 9,12-octadecadienoic acid methyl ester (linoleic acid) not only offers a range of health benefits, such as anti-inflammatory, anticancer, and hypocholesterolemic properties, but also demonstrates the utility of these byproducts due to its composition of antioxidants. The 9-octadecenoic methyl ester (Z) [41,42] is similarly linked to antioxidant and anticancer activities.

These findings highlight the potential health benefits of these compounds, underscoring the value of industrial annatto residue.

#### 3.1.3. Dichloromethane-Soluble Compounds

When investigating the presence of dichloromethane-soluble compounds in sample FS of the industrial annatto residue, *springene (isomer)* and 1,5,9-Cyclotetradecatriene, 1,5,9-trimethyl-12-(1-methylethenyl), or Cembrene were identified (chromatogram in the Supplementary Materials). Cembrenes are biosynthesized by macrocyclization of geranylgeranyl pyrophosphate [43]. 1,5,9-Cyclotetradecatriene, 1,5,9-trimethyl-12-(1-methylethenyl)- is a diterpene, a natural product found in *Toona calantas* [44] and in Japanese domestic tobacco, *Nicotiana tabacum* cv. *Suifu* [45].

In the search for dichloromethane-soluble compounds in sample TT of the industrial annatto residue, *springene (isomers)* was identified (chromatogram in the Supplementary Materials).

The isomers *α* and *β-springene* were identified in the present work in the dilutions made in hexane (see Section 3.1.1), and *α-springene* was also identified in the dilutions made in methanol (see Section 3.1.2).

Table 3 shows the identification by GC-MS of the dichloromethane-soluble compounds present in samples FS and TT of the industrial annatto residue.

#### 3.1.4. HS-SPME-GC-MS

In order to identify the volatile compounds obtained by HS-SPME-GC-MS, the mass spectra were compared with the data found in the NIST library (chromatograms in the Supplementary Materials), and confirmation with standards was obtained when possible (Table 4 and Table 5).

In the search for the volatile compounds present in sample FS of the annatto byproduct, the presence of the compounds *p*-xylene; 6-methyl-5-hepten-2-one; 5,9-undecadien-2-one; 6,10-dimethyl; and farnesal was confirmed by comparison with their standards.

In the search for the volatile compounds present in sample TT of the annatto byproduct, the presence of the compounds *p*-xylene; *o*- xylene; 5-hepten-2-one,6-methyl; 5,9-undecadien-2-one,6,10-dimethyl-, (E); and cis-β-farnesene was confirmed by comparison with their authentic standards.

The experimental molecular distillation conditions were planned for the maximum recovery yield of geranylgeraniol and δ-tocotrienol. The residence time in the molecular distiller was probably insufficient for the total removal of these volatile compounds from the annatto byproduct, and their presence was unknown up to the present moment. The thermal degradation compounds of bixin could be formed throughout the distillation process, since it occurred at high temperatures. The compounds might only be present at trace levels and still be identified by HS-SPME-GC-MS, which is a highly sensitive technique.

The volatile compounds could be formed from multiple precursor nutrients, such as fatty acids, amino acids, and carotenoids [46,47], and volatile derivatives from carotenoids exert a highly significant contribution to the odor of fruits and flowers. The compounds 6-methyl-5-hepten-2-one and 5,9-undecadien-2-one, 6,10-dimethyl-, (E), which are formed during the metabolism of carotenoids in fruits [48,49], were the most abundant volatile compounds identified in samples FS and TT (Table 4 and Table 5), and the occurrence of both was described in annatto leaf and seed extracts [6]. 6-methyl-5-hepten-2-one was also reported in commercial annatto residues [50]. The repellent action of three aldehydes, octanal, nonanal, and decanal, and of the ketones 6-methyl-5-hepten-2-one and 5,9-undecadien-2-one, 6,10-dimethyl-, €, was tested in different concentrations for the vectors *Anopheles gambiae*, *Culex quinquefasciatus,* and *Aedes aegypti*, which are considered the most important in disease propagation [51]. The mixture of the two ketones in a 1:1 proportion was highly efficient against all the vectors studied, exceeding the repellence of low concentrations of the synthetic compound N, N-diethyl-m-toluamide (DEET). DEET is the most widely used synthetic insect repellent and is considered the “golden standard” repellent, providing lasting protection for up to 8 h after application [52].

Mckeown, in ref. [53], described the thermal degradation mechanism of bixin, forming aromatic compounds such as toluene, *m*-xylene, *o*-xylene, *m*-toluic acid, and *m*-toluic acid, methyl ester. The same author stated that naphthalene derivatives could also be formed, but this was not confirmed. The presence of compounds such as toluene and m and o-xylenes in annatto products is frequently cited in the literature, and *m*-xylene is the principal volatile compound produced when bixin is decomposed [53,54,55].

Some research projects have concentrated on determining the levels of these volatile compounds in the annatto products, as well as testing solvents and conditions to recover the pigment from annatto seeds with less formation of these compounds. Vegetable oils, alkaline solutions, and acetone are the most promising solvents due to their availability and low human health risks as compared to the others [55,56,57,58].

### 3.2. UPLC-MS/QTOF

#### 3.2.1. Sample FS

Table S1 and Figure S11 (Supplementary Materials) highlight the identification of bioactive non-volatile compounds in the FS sample of industrial annatto residue, emphasizing its robust antioxidant composition. Among these, dihydroactinidiolide (RT 6.83 min; [M-H]^+^ 181.124 Da), a terpenoid lactone with the molecular formula C_11_H_16_O_2_, is a carotenoid oxidation product known for its antioxidant relevance. This compound has been reported in *Actinidia polygama* leaves [59], white teas [60], green and black teas [61,62], *Rooibos* tea [63], and *Arabidopsis* leaves [64]. Similarly, dihydrochalcone, 3-phenylpropiophenone (RT 7.94 min; [M-H]^+^ 211.1143 Da), a dihydrochalcone (C_15_H_14_O) biosynthesized via the phenylpropanoid pathway, exhibits potent phenolic antioxidant properties and has been identified in apples [65], cranberries [66], and herbal teas [67]. Another significant compound, 2,3-dihydroxy-N-[(2S,3S,4R,8E)-1,3,4-trihydroxy-8-octadecen-2-yl]tetracosanamide (RT 12.72 min; [M-H]^+^ 698.629 Da), a ceramide (C_42_H_83_NO_6_), is associated with lipid-based antioxidant activity and has been detected in *Helianthus annuus* seeds [68], *Acanthopanax gracilistylus* [69], and *Lupinus luteus* seeds [70]. Lastly, 1,3-dilinolein (RT 12.96 min; [M-H]^+^ 617.5123 Da), a diacylglycerol (C_39_H_68_O_5_), is notable for its antioxidant properties and health benefits, such as triglyceride reduction and fat metabolism [71,72]. Collectively, these compounds underline the potential of industrial annatto residue as a valuable source of natural antioxidants, with applications in food, cosmetics, and sustainable biopackaging development.

#### 3.2.2. Sample TT

Table S2 shows the identification data of the non-volatile compounds obtained by UPLC-QTOF present in sample TT of the industrial annatto residue (replicas 1, 2, and 3) for the positive mode, and Figure S12 shows the chromatogram (A, positive mode; B, negative mode) in the Supplementary Materials.

The identification proposed for the compound with an RT of 8.13 min and observed [M-H]^+^ of 316.2862 Da was halisphingosine A, with a molecular formula of C_18_H_37_ NO_3_, previously described in *foeniculum vulgare* seeds [73].

The compound with an RT of 8.35 min, observed [M-H]^+^ of 328.2855 Da, and molecular formula of C_19_H_37_NO_3_ showed fragmentation coinciding with amino acid derivatives (palmitoyllanine; N-acyl-L-amino acid; palmitoyl sarcosine; N-tetradecanoyl- valine and ethyl N-acetyl-N-dodecyl-β-alaninate), but there are no reports in the literature citing the occurrence of any of these compounds in products derived from annatto. The presence of the amino acids valine, isoleucine, and alanine was described in annatto seed bran [74].

The identification proposed for the compound with an RT of 9.57 min and observed [M-H]^+^ of 321.2432 Da was icomucret, with a molecular formula of C_20_H_32_O_3_. This compound can be produced from the auto-oxidation of arachidonic acid [75]. Arachidonic acid was previously described in annatto seed oil by Costa et al. [76].

The identification proposed for the compound with an RT of 10.04 min and observed [M-H]^+^ of 323.2592 Da was 2,6,16-kauranetriol, which is a diterpene. No reports of the presence of this compound in annatto seeds were found in the literature.

The compound with an RT of 10.44 min, observed [M-H]^+^ of 564.5349 Da, and molecular formula of C_36_H_69_NO_3_ showed fragmentation coinciding with ceramide derivatives (ceramide (d18:1/9Z-18:1); N-oleoyl-D- sphingosine; (9E)-N-[(2S,4E)-1,3-dihydroxy-4-octadecen-2-yl]-9-octadecenamid; (9E,12E)-N-(1,3-dihydroxy-2-octadecanyl)-9,12-octadecadienamide). The ceramides are a family of lipid molecules composed of sphingosine plus a fatty acid and are found in high concentrations in the cell membrane [77]. Ceramides were identified in sunflowers, *Helianthus annuus* L. [68], and in the plants *Acanthopanax gracilistylus* [78] and *Stropharia rugosoannulata* [79]. No reports of the presence of ceramides in annatto seeds were found in the literature.

The identification proposed for the compound with an RT of 11.51 min and observed [M-H]^+^ of 321.2432 Da was icomucret, with a molecular formula of C_20_H_32_O_3_. This compound can be produced from the auto-oxidation of arachidonic acid and is the same compound identified with an RT of 9.57.

The identification proposed for the compound with an RT of 12.56 min and observed [M-H]^+^ of 545.5092 Da was phytoene; 15-cis-phytoene, with a molecular formula of C_40_H_64_, a carotenoid previously identified in annatto [80]. The biosynthesis of carotenoids starts with the condensation of two molecules of geranylgeranyl pyrophosphate to produce cis-phytoene (C40), which is the base structure of all carotenoids. The cis-phytoene then passes through desaturation reactions to synthesize lycopene [81], which is the simplest carotenoid molecule (linear molecule with 40 carbon atoms) and is the substrate for the generation of most of the cyclical carotenoids by way of cyclic, hydroxylation, or oxidation reactions or a combination of these three reactions. These reactions occur at one of the extremities of the lycopene chain, forming distinct carotenoids by way of this biosynthetic route [82,83].

The identification proposed for the compound with an RT of 13.22 min and observed [M-H]^+^ of 573.4656 Da was kitol, with a molecular formula of C_40_H_60_O_2_. Kitol is a vitamin A dimer whose structure was previously elucidated by mass spectrometry [84]. Kitols are formed by chemical reactions between retinol and retinyl ester molecules [85,86]. Kitol was first reported in whale liver oil [87]. Dimers can be formed synthetically in thermally induced Diel–Alder reactions [86]. Kitol may have been formed during the production process, which uses high temperatures, and due to the abundant occurrence of vitamin a derivative in annatto seeds.

### 3.3. Antioxidant Capacity and Active Package

In the determination of the antioxidant activity of the industrial annatto residues, considerable variation was found between the results. Samples Batch 1 to 4 showed little or no activity, whereas samples Batch 5, TT, and FS showed very strong or medium antioxidant activity as compared to the blanks (Table 6).

After evaluating the antioxidant activity of the industrial annatto residue samples, active films were prepared with samples Batch 5, TT, and FS.

Table 6 shows the results obtained for the evaluation of the antioxidant activity of the active films prepared with samples TT, FS, and Batch 5.

This activity was notably preserved in the packaging films produced with samples FS and TT, yielding a packaging material with distinct antioxidant properties. In an unprecedented advancement, we proposed an innovative active packaging material derived from industrial annatto seed residues. This material exhibited a striking orangey hue, attributed to the pigments inherent in annatto. The findings outlined in this report could hold significant technological value for industries seeking novel, sustainable applications for agro-industrial residues.

Natural antioxidants are recognized for their ability to inhibit the formation of reactive species or neutralize them before they can harm food processes. According to the literature [7], the observed antioxidant properties can be attributed to a variety of bioactive volatile compounds. Notably, the antioxidant properties of active extracts are not merely the result of their primary constituents. Minor components may contribute synergistically, enhancing overall antioxidant activity through compound interactions [7].

Analytical studies confirm that annatto byproducts are rich in bioactive compounds with well-documented antioxidant properties. As such, industrial annatto residues represent an untapped and valuable resource for the development of active biopackaging films with enhanced functionality.

The integration of plant-based active ingredients into packaging materials exemplifies a pivotal shift towards a circular economy, where industrial residues are transformed into valuable resources. Researchers have increasingly focused on this approach [88,89] as it not only repurposes agro-industrial byproducts but also reduces waste and minimizes reliance on non-renewable resources. The innovation presented in this work directly addresses consumer demand for sustainable solutions, promoting the development of eco-friendly products that align with global efforts to create closed-loop systems and foster resource efficiency.

## 4. Conclusions

The chemical composition of industrial annatto residue reveals a diverse array of bioactive compounds, several of them with antioxidant properties. To leverage the industrial annatto residue for the development of dynamic packaging solutions, it is essential to extract its oil fraction. This strategic separation enables the effective incorporation of the residue into active packaging systems. Such valorization aligns with the principles of the circular economy, where waste streams from agro-industrial processes are redirected toward high-value applications. By adopting this approach, the annatto byproduct offers significant technological potential, especially for industries committed to sustainable innovation and resource efficiency. The integration of this residue into circular practices not only minimizes environmental impact but also creates social and economic benefits, transforming a once-discarded material into a valuable contributor to the bioeconomy.

## Figures and Tables

**Table 1:** Identification by GC-MS of the alkanes and hexane-soluble compounds present in the annatto byproducts.

pk	RT min	RI	Compounds	Molecular Formula	CAS	Match	Samples						
							TT	FS	Batch 1	Batch 2	Batch 3	Batch 4	Batch 5
1	19.262	1693	Cyclododecane	C_12_H_24_	294-62-2	92	X						
2	21.675	1925	Springene (isomer)	C_20_H_32_	70901-63-2	95	X	X	X	X	X	X	X
3	21.986	1947	*E*(*β*)-Farnesene	C_20_H_32_	1000432-20-1	86	X	X	X	X	X	X	X
4	22.139	1972	Springene (isomer)	C_20_H_32_	77898-97-6	91	X	X	X	X	X	X	X
5	22.352	1994	Springene (isomer)	C_20_H_33_	70901-63-2	90	X			X			
6	22.394	1998	Eicosane	C_20_H_42_	112-95-8	96	X						
8	23.696	2140	Linoelaidic acid	C_18_H_32_O_2_	506-21-8	99	X	X	X		X	X	X
9	23.742	2145	9,12-Octadecadienoic acid (Z,Z)-	C_18_H_32_O_2_	60-33-3	97	X	X	X	X	X	X	X
10	23.959	2170	Octadecanoic acid	C_18_H_32_O_2_	57-11-4	93		X	X	X	X	X	X
11	24.218	2199	2,6,10-Dodecatrien-1-ol, 3,7,11-trimethyl	C_15_H_26_O	4602-84-0	91		X	X		X	X	X
12	24.222	2199	Trans-Geranylgeraniol	C_20_H_34_O	24034-73-9	97	X		X		X		
13	24.306	2209	1-Octadecene	C_18_H_36_	112-88-9	94				X			
14	24.435	2224	Trans-Farnesol	C_15_H_26_O	106-28-5	86	X		X				X
16	25.066	2298	Tricosane	C_23_H_48_	638-67-5	94	X	X	X	X	X	X	
17	25.667	2371	9-Octadecenamide, (Z)-	C_18_H_35_NO	301-02-0	99	X	X	X	X	X	X	X
18	25.792	2386	Octadecanoic acid, butyl ester	C_22_H_44_O_2_	123-95-5	93	X	X		X	X	X	
19	25.888	2398	Tetracosane	C_24_H_50_	646-31-1	98	X	X	X	X	X	X	
20	25.976	1998	3-Eicosene, (E)-	C_20_H_40_	74685-33-9	96	X			X		X	
21	26.143	2430	Ethanol,2-(9-octadecenyloxy)-(Z)	C_20_H_40_O_2_	5353-25-3	86	X						
23	26.673	2498	Pentacosane	C_25_H_52_	626-99-2	97	X	X	X	X	X	X	
24	27.433	2598	Hexacosane	C_26_H_54_	630-01-3	90	X	X	X	X	X	X	
25	28.164	2698	Heptacosane	C_27_H_56_	593-49-7	97	X			X	X	X	
26	28.865	2797	Octacosane	C_28_H_58_	630-02-4	97	X	X	X	X	X	X	
27	29.55	2897	Nonacosane	C_29_H_60_	630-03-5	98	X	X	X	X	X	X	
28	30.217	2998	Triacontane	C_30_H_62_	638-68-6	99	X	X	X	X	X	X	
29	30.861	3088	δ-Tocotrienol	C_27_H_40_O_2_	25612-59-3	97	X	X	X	X	X	X	X
30	30.932	3098	Hentriacontane	C_31_H_64_	630-04-6	99	X	X	X	X			
31	31.739	3198	Dotriacontane	C_32_H_66_	544-85-4	98		X	X				
32	32.665	3298	Tritriacontane	C_33_H_68_	630-05-7	97		X					

RT: retention time. RI: retention index calculated for the HP-5MS capillary column (30 m × 0.25 µm × 250 µm). *n* = 3. Letter “X” means that compound is present in the sample.

**Table 2:** Identification by GC-MS of the methanol-soluble compounds present in the annatto byproducts.

RT	RI	Compounds	Molecular Formula	CAS	Match
Sample FS					
22.370	1972	Springene (isomer)	C_20_H_32_	77898-97-6	98
23.535	2098	9,12-Octadecadienoic acid, methyl ester	C_19_H_34_O_2_	2462-85-3	99
23.588	2101	9-octadecenoic (Z), methyl ester	C_19_H_36_O_2_	112-62-9	99
Sample TT					
22.369	1972	Springene (isomer)	C_20_H_32_	77898-97-6	91
22.59	1995	Springene (isomer)	C_20_H_32_	77898-97-6	86
23.554	2098	9,12-Octadecadienoic acid, methyl ester	C_19_H_34_O_2_	2462-85-3	99

RT: retention time. RI: retention index calculated for the HP-5MS capillary column (30 m × 0.25 µm × 250 µm). *n* = 3.

**Table 3:** Identification by GC-MS of the dichloromethane-soluble compounds present in the annatto byproducts.

RT	RI	Compounds	Molecular Formula	CAS	Match
Sample FS					
22.375	1972	Springene (isomer)	C_20_H_32_	77898-97-6	94
22.464	1982	1,5,9-Cyclotetradecatriene, 1,5,9-trimethyl-12-(1-methylethenyl)	C_20_H_32_	038748-84-4	89
Sample TT					
17.249	1502	β-Cadinene	C_15_H_24_	523-47-7	96
21.864	1920	Springene (isomer)	C_20_H_32_	70091-63-2	93
22.355	1971	Springene (isomer)	C_20_H_32_	77898-97-6	95
22.574	1994	Springene (isomer)	C_20_H_32_	77898-97-6	90

RT: retention time. RI: retention index calculated for the HP-5MS capillary column (30 m × 0.25 µm × 250 µm). *n* = 3.

**Table 4:** Identification by HS-SPME-GC-MS of the volatile compounds present in sample FS of the annatto byproducts.

RT (min)	RI	Compound	Molecular Formula	CAS	Match
3.599		Toluene	C_7_H_8_	108-88-3	92
6.192		*p*-xylene	C_8_H_10_	106-42-3	97
6.719		Ethanone, 1-(1-cyclohexen-1-yl)	C_8_H_12_O	932-66-1	90
7.032		3-Methylcyclopentyl acetate	C_8_H_14_O_2_	24070-70-0	86
9.260	988.0	6-Methyl-5-hepten-2-one	C_8_H_14_O	110-93-0	95
9.649	1005.8	2,4-Heptadienal,(E,E)	C_7_H_10_O	4313-03-5	91
10.597	1055.8	2-Octenal, (E)	C_8_H_14_O	2548-87-0	87
11.061	1080.2	Benzaldehyde, 4-methyl	C_8_H_8_O	104-87-0	90
11.190	1087.0	Ethyl 2-(5-methyl-5-vinyltetrahydrofuran-2-yl) propan-2-yl carbonate	C_13_H_22_O_4_	1000373-80-3	91
11.240	1089.6	2-Nonanone	C_9_H_18_O	821-55-6	90
11.470	1102.0	6-Methyl-3,5-heptadiene-2-one	C_8_H_12_O	1604-28-0	94
12.497	1163.0	Isoneral	C_10_H_16_O	1000414-18-0	96
12.785	1181.5	3,6-Octadienal,3,7-Dimethyl	C_10_H_16_O	55722-59-3	95
13.073	1198.9	Dodecane	C_10_H_16_O	112-40-3	97
13.157	1204.4	Benzoic acid, 4-methyl-, methyl ester	C_9_H_10_O_2_	99-75-2	97
13.370	1218.8	5-Isopropenyl-2-methylcyclopent-1-enecarboxaldehyde	C_10_H_14_O	1000190-36-8	95
13.432	1222.9	1-Cyclohexene-1-carboxaldehyde, 2,6,6-trimethyl	C_10_H_16_O	432-25-7	98
13.583	1233.1	Oxiranecarboxaldehyde, 3-methyl-3-(4-methyl-3-pentenyl)	C_10_H_16_O_2_	16996-12-6	90
13.704	1241.3	Neral	C_10_H_16_O	106-26-3	96
13.925	1256.1	Benzene, 1,3-bis(1,1-dimethylethyl)	C_14_H_22_	1014-60-4	96
14.150	1271.3	2,6-Octadienal, 3,7-dimethyl-, (E)	C_10_H_16_O	141-27-5	97
14.188	1273.8	4,8-Dimethylnona-3,8-dien-2-one	C_11_H_18_O	872858-42-9	97
14.288	1280.6	Pentacosane	C_25_H_52_	629-99-2	86
14.468	1292.7	2-Undecanone	C_11_H_22_O	112-12-9	94
15.178	1344.0	Cyclohexene, 4-ethenyl-4-methyl-3-(1-methylethenyl)-1-(1-methylethyl)-, (3R-trans)	C_15_H_24_	20307-84-0	99
15.353	1356.8	alpha-Cubebene	C_15_H_24_	17699-14-8	99
16.635	1454.2	5,9-Undecadien-2-one, 6,10-dimethyl-,(E)	C_13_H_22_O	3796-70-1	94
16.706	1459.7	Zonarene	C_15_H_24_	41929-05-9	89
17.115	1491.8	trans-β-Ionone	C_13_H_20_O	79-77-6	98
17.215	1499.6	Naphthalene, decahydro-4a-methyl-1-methylene-7-(1-methylethenyl)-, [4aR-(4aα,7α,8aβ)]-	C_15_H_24_	17066-67-0	97
17.520	1525.0	Naphthalene, 1,2,3,4,4a,5,6,8a-octahydro-7-methyl-4-methylene-1-(1-methylethyl)-, (1α,4aβ,8aα)-	C_15_H_24_	39029-41-9	99
17.637	1534.8	3,5,9-Undecatrien-2-one, 6,10-dimethyl-, (E,Z)-	C_13_H_20_O	13927-47-4	72
17.733	1542.8	2(4H)-Benzofuranone, 5,6,7,7a-tetrahydro-4,4,7a-trimethyl-, (R)-	C_11_H_16_O_2_	17092-92-1	98
17.800	1548.4	Naphthalene, 1,2,4a,5,6,8a-hexahydro-4,7-dimethyl-1-(1-methylethyl)-, [1S-(1α,4aβ,8aα)]	C_15_H_24_	24406-05-1	99
18.873	1640.1	β-Guaiene	C_15_H_24_	88-84-6	91
19.691	1712.7	Eicosane	C_20_H_42_	112-95-8	90
20.071	1748.0	Farnesal	C_15_H_24_O	19317-11-4	97
20.622	1799.2	Octadecane	C_18_H_38_	593-45-3	97
21.875	1922.1	trans-Geranilgeraniol	C_20_H_34_O	7614-21-3	87
21.929	1927.6	5,9,13-pentadecatrien--2-one, 6,10,14-Trimethyl-	C_18_H_30_O	762-29-8	93

RT: retention time. RI: retention index calculated for the HP-5MS capillary column (30 m × 0.25 µm × 250 µm). *n* = 3.

**Table 5:** Identification by HS-SPME-GC-MS of the volatile compounds present in sample TT of the annatto byproducts.

RT (min)	RI	Compounds	Molecular Formula	CAS	Match
3.600		Toluene	C_7_H_8_	108-88-3	92
5.967		3-Penten-1-ol,4-methyl-	C_6_H_12_O	763-89-3	86
6.226		p-xylene	C_8_H_10_	106-42-3	97
6.656		o-xylene	C_8_H_10_	95-47-6	94
6.827		m-xylene	C_8_H_10_	108-38-3	97
7.044		3-Methylcyclopentyl acetate	C_8_H_14_O_2_	24070-70-0	86
8.547	957.1	Benzaldehyde	C_7_H_6_O	100-52-7	97
9.194	985.1	6-Methyl-5-hepten-2-one	C_8_H_14_O	110-93-0	97
9.386	993.4	2,4-Heptadienal, (E,E)-	C_7_H_10_O	4313-03-5	87
9.654	1006.1	2,4-Heptadienal	C_7_H_10_O	5910-85-0	87
10.042	1026.5	D-Limonene	C_10_H_16_	5989-27-5	98
10.798	1066.4	Benzaldehyde, 2-methyl-	C_8_H_8_O	529-20-4	96
11.240	1089.6	2-Nonanone	C_9_H_18_O	67801-33-6	90
11.391	1097.6	Linalool	C_10_H_18_O	78-70-6	97
11.462	1101.5	6-Methyl-3,5-heptadiene-2-one	C_8_H_12_O	1604-28-0	94
12.460	1161.9	Benzenemethanol, α,4-dimethyl-	C_9_H_12_O	536-50-5	95
12.652	1173.5	Ethanone, 1-(3-methylphenyl)-	C_9_H_10_O	585-74-0	97
12.756	1179.7	Benzene, 1-methyl-4-(1-methyl-2-propenyl)-	C_11_H_14_	97664-18-1	91
12.835	1184.5	Ethanone, 1-(4-methylphenyl)-	C_9_H_10_O	122-00-9	97
13.148	1203.8	Decanal	C_10_H_20_O	112-31-2	97
13.428	1222.7	beta-Cyclocitral	C_10_H_16_O	432-25-7	97
13.712	1241.8	Neral	C_10_H_16_O	106-26-3	96
13.950	1257.8	1H-Indene, 1,3-dimethyl-	C_11_H_12_	2177-48-2	96
14.046	1264.3	1H-Indene, 4,7-dimethyl-	C_11_H_12_	6974-97-6	96
14.134	1270.2	2,6-Octadienal, 3,7-dimethyl-, (E)-	C_10_H_16_O	141-27-5	97
14.184	1273.6	4,8-Dimethylnona-3,8-dien-2-one	C_11_H_18_O	872858-42-9	97
14.764	1313.7	1,2,3-Trimethylindene	C_12_H_14_	4773-83-5	96
15.173	1343.6	Cyclohexene, 4-ethenyl-4-methyl-3-(1-methylethenyl)-1-(1-methylethyl)-, (3R-trans)-	C_15_H_24_	20307-84-0	99
15.353	1356.8	alpha-Cubebene	C_15_H_24_	17699-14-8	99
15.449	1363.8	Naphthalene, 1,2,3,4-tetrahydro-1,1,6-trimethyl-	C_13_H_18_	475-03-6	96
16.050	1408.4	1H-Inden-1-one, 2,3-dihydro-3,4,7-trimethyl-	C_12_H_14_O	35322-84-0	93
16.109	1413.0	Naphthalene, 2,6-dimethyl-	C_12_H_12_	581-42-0	98
16.351	1431.9	Naphthalene, 1,5-dimethyl-	C_12_H_12_	571-61-9	98
16.652	1455.5	5,9-Undecadien-2-one, 6,10-dimethyl-, (E)-	C_13_H_22_O	3796-70-1	94
16.785	1465.9	cis-β-Farnesene	C_15_H_24_	28973-97-9	90
17.061	1487.5	Isoledene	C_15_H_24_	95910-36-4	94
17.111	1491.5	trans-β-Ionone	C_13_H_20_O	79-77-6	98
17.520	1525.0	Naphthalene, 1,2,3,4,4a,5,6,8a-octahydro-7-methyl-4-methylene-1-(1-methylethyl)-, (1α,4aβ,8aα)	C_15_H_24_	39029-41-9	99
17.608	1532.4	Naphthalene, 1,2,3,5,6,8a-hexahydro-4,7-dimethyl-1-(1-methylethyl)-, (1S-cis)-	C_15_H_24_	483-76-1	95
17.654	1536.2	3,5,9-Undecatrien-2-one, 6,10-dimethyl-, (E,Z)-	C_13_H_20_O	13927-47-4	93
17.720	1541.7	2(4H)-Benzofuranone, 5,6,7,7a-tetrahydro-4,4,7a-trimethyl-, (R)-	C_11_H_16_O_2_	17092-92-1	95
17.800	1548.4	Naphthalene, 1,2,4a,5,6,8a-hexahydro-4,7-dimethyl-1-(1-methylethyl)-, [1S-(1α,4aβ,8aα)]-	C_15_H_24_	24406-05-1	98
17.870	1554.3	α-Calacorene	C_15_H_20_	21391-99-1	93
18.025	1567.2	1,6,10-Dodecatrien-3-ol, 3,7,11-trimethyl-	C_15_H_26_O	40716-66-3	87
18.088	1572.5	1,2,3,4-Tetrahydro-8-methyl-1-naphthalenemethanol	C_12_H_16_O	36052-28-5	92
18.284	1588.8	3,5,9-Undecatrien-2-one, 6,10-dimethyl-, (E,E)-	C_13_H_20_O	3548-78-5	93
18.405	1598.9	Hexadecane	C_16_H_34_	544-76-3	96
18.881	1640.8	Isospathulenol	C_15_H_24_O	88395-46-4	96

RT: retention time. RI: retention index calculated for the HP-5MS capillary column (30 m × 0.25 µm × 250 µm). *n* = 3.

**Table 6:** Results obtained for antioxidant activity of the annatto byproducts and active films.

	Sample	Mean	SD	RSD%	Result
Annatto byproducts	Batch 1	100			No CAOX
	Batch 2	100			No CAOX
	Batch 3	74	2	2	Weak CAOX
	Batch 4	87	7	8	Weak CAOX
	Batch 5	60	2	3	Medium CAOX
	TT	42	1	3	Very strong CAOX
	FS	45	3	6	Very strong CAOX
Active films prepared with annatto byproducts	Batch 5	100			No CAOX
	TT	55	4	8	Very strong CAOX
	FS	70	5	7	Medium CAOX

*n* = 3.

## Data Availability

The original contributions presented in the study are included in the article and in the Supplementary Material, further queries can be directed to the corresponding authors.

## Supplementary Materials

The following supporting information can be downloaded at https://www.mdpi.com/article/10.3390/foods14040704/s1: Figure S1: Chromatogram of the C7–C40 alkane standards; Figure S2: Chromatogram of sample Batch 2, industrial annatto residue, added to a solution of the C7–C40 alkane standards; Figure S3: Chromatogram of sample TT—hexane obtained by CG-MS; Figure S4: Chromatogram of sample FS—hexane obtained by CG-MS; Figure S5: Chromatogram of sample FS—methanol obtained by CG-MS; Figure S6: Chromatogram of sample TT—methanol obtained by CG-MS. Figure S7: Chromatogram of sample FS—dichloromethane obtained by CG-MS; Figure S8: Chromatogram of sample TT—dichloromethane obtained by CG-MS; Figure S9: Chromatogram of sample FS (volatile compounds) obtained by HS-SPME-GC-MS; Figure S10: Chromatogram of sample TT (volatile compounds) obtained by HS-SPME-GC-MS; Figure S11: Chromatogram of sample FS (non-volatile compounds) obtained by UPLC-QTOF (A, positive mode; B, negative mode); Figure S12: Chromatogram of sample TT (non-volatile compounds) obtained by UPLC-QTOF (A, positive mode; B, negative mode); Table S1: Identification data of the non-volatile compounds obtained by UPLC-QTOF present in sample FS of the industrial annatto residue (replicas 1, 2, and 3); Table S2: Identification data of the non-volatile compounds obtained by UPLC-QTOF present in sample TT of the industrial annatto residue (replicas 1, 2, and 3).
