# Supplementary material for: Promoting Circular Economy by Leveraging Annatto Byproducts from Bixa orellana L. into Sustainable Antioxidant Food Packaging
Source: Foods. 2025 Feb 19;14(4):704. doi: 10.3390/foods14040704 (PMC11854884; doi:10.3390/foods14040704)
Supplement: Supplementary file 1 [file foods-14-00704-s001.zip › foods-3411800-supplementary.pdf]

Supplementary materials for:

Promoting Circular Economy by Leveraging Annatto
Byproducts from *Bixa orellana* L. into Sustainable Antioxidant Food Packaging

Vanilda Aparecida Soares de Arruda-Peixoto [1,*], Paula Vera Estacho [2], Magdalena Wrona [3], Paulo Roberto Nogueira Carvalho [1], Roseli Aparecida Ferrari [1], Cristina Nerin [2] and Elena Canellas [2,*]

[1] Food Technology Institute, Av. Brasil. 2280, Campinas 13070-178, SP, Brazil; carvalho@ital.sp.gov.br (P.R.N.C.); roseliferrari@ital.sp.gov.br (R.A.F.)

[2] Department of Analytical Chemistry, Aragon Institute of Engineering Research I3A, EINA—University of Zaragoza, Torres Quevedo Building, María de Luna 3, 50015 Zaragoza, Spain; pvera@unizar.es (P.V.E.); magdalena.wrona@unizar.es (M.W.); cnerin@unizar.es (C.N.)

[3] Institute of Bio- and Geosciences, 2, Forschungszentrum Jülich GmbH, 52428, Jülich, Germany
m.wrona@fz-juelich.de (M.W.)

* Correspondence: cravoecanela.sp@alumni.usp.br (V.A.S.d.A.-P.); elenac@unizar.es (E.C.A.);
Tel.: +55-019-3743-1774 (V.A.S.d.A.-P.); +34-876-55-50-49 (E.C.A.)

Academic Editor(s): Name

Received: 20 December 2024
Revised: 5 February 2025
Accepted: 8 February 2025
Published: date

Citation: Arruda-Peixoto, V.A.S.d.; Vera Estacho, P.; Wrona, M.; Nogueira Carvalho, P.R.; Aparecida Ferrari, R.; Nerin, C.; Canellas, E. Promoting Circular Economy by Leveraging Annatto Byproducts from *Bixa orellana* L. into Sustainable Antioxidant Food Packaging. *Foods* **2025**, *14*, x. https://doi.org/10.3390/xxxxx

Copyright: © 2025 by the authors. Submitted for possible open access publication under the terms and conditions of the Creative Commons Attribution (CC BY) license (https://creativecommons.org/licenses/by/4.0/).

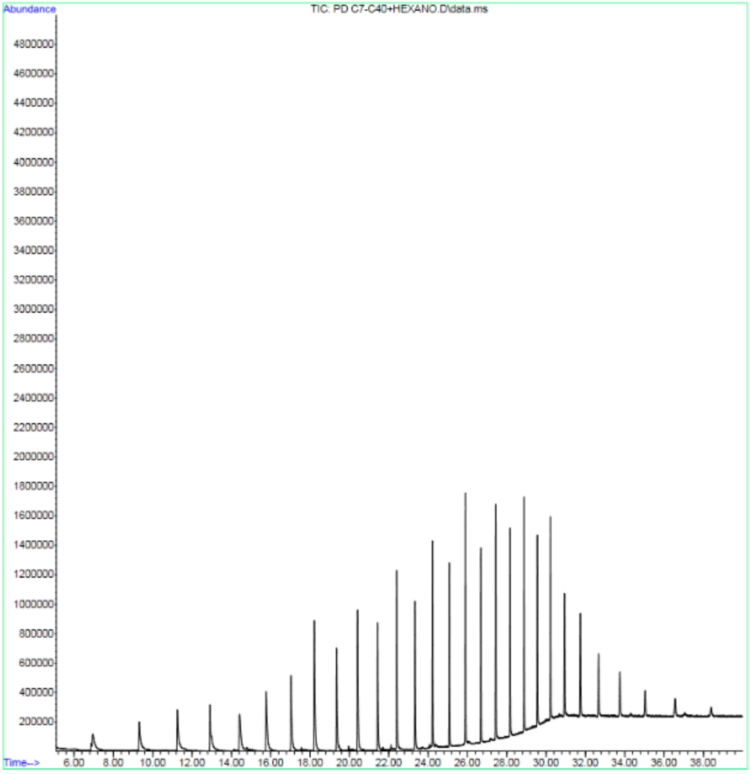

Figure S1- *Chromatogram of the C7-C40 alkane standards*

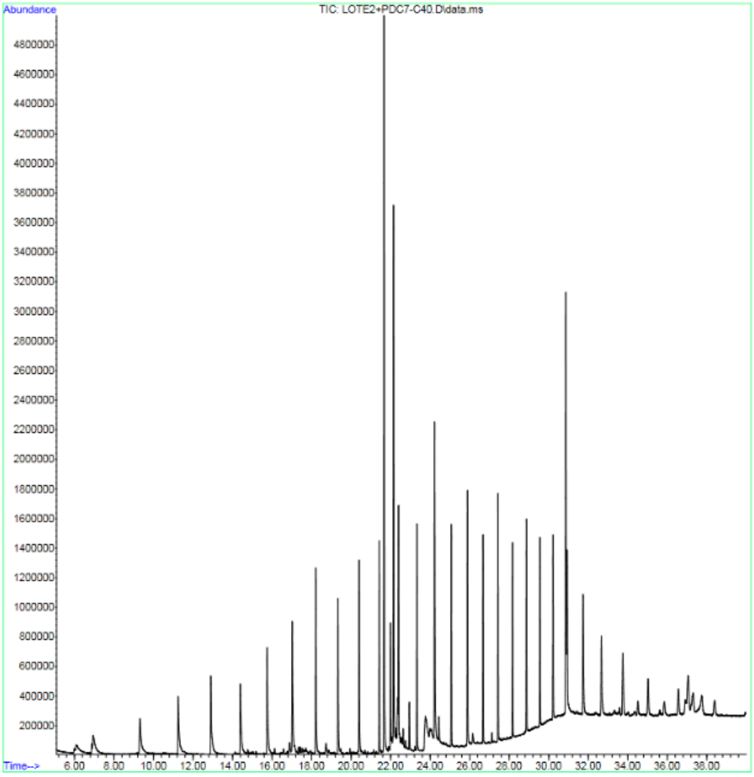

Figure S2 - *Chromatogram of sample Bacth 2, industrial annatto residue, added to a solution of the C7-C40 alkane standards.*

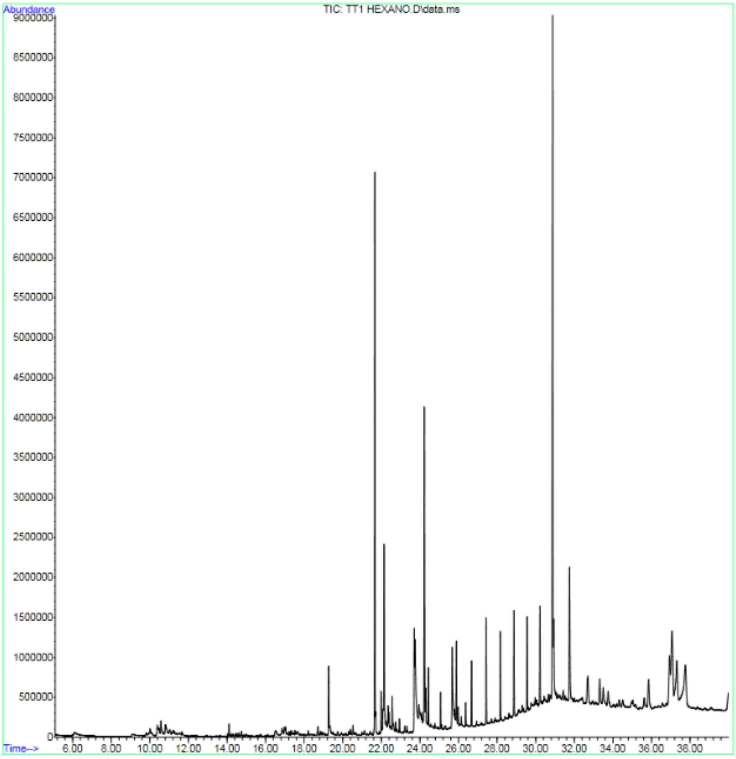

Figure S3 - *Chromatogram of sample TT – hexane, obtained by CG-MS*

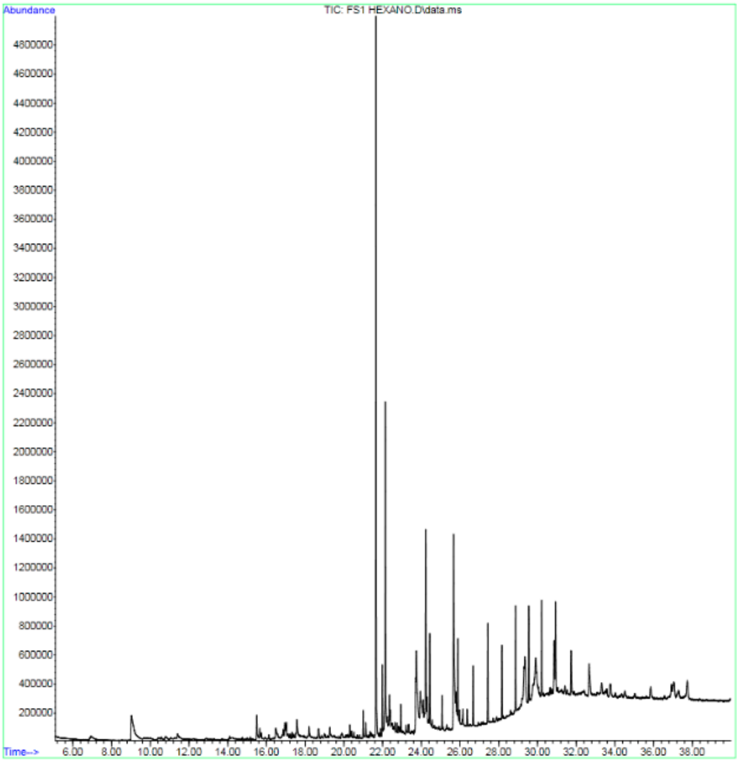

Figure S4 - *Chromatogram of sample FS – hexane, obtained by CG-MS*

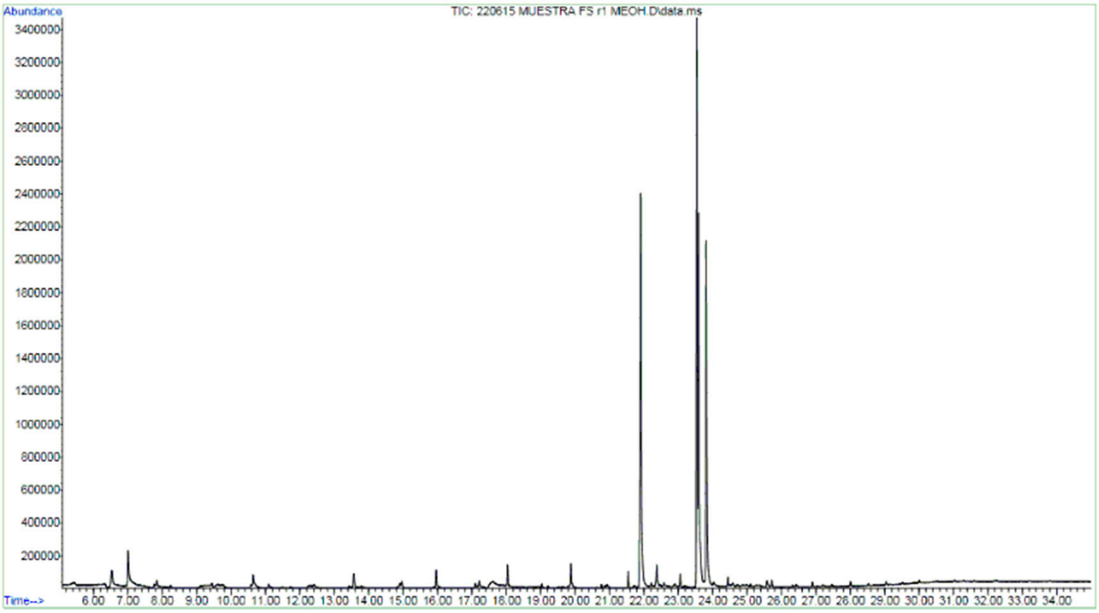

Figure S5 *- Chromatogram of sample FS - methanol, obtained by CG-MS*

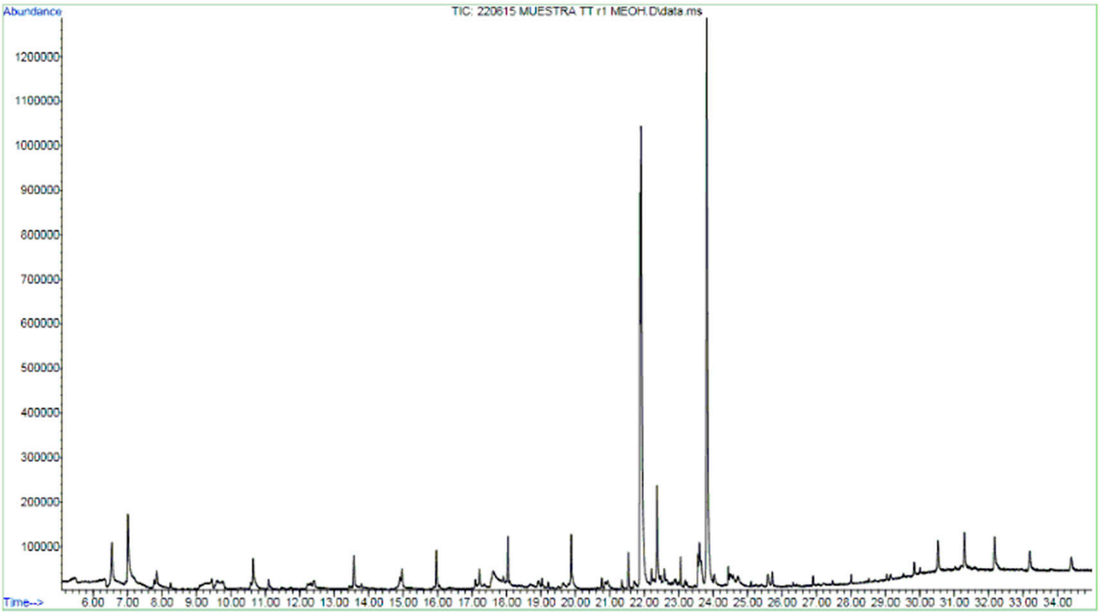

Figure S6 *- Chromatogram of sample TT- methanol, obtained by CG-MS*

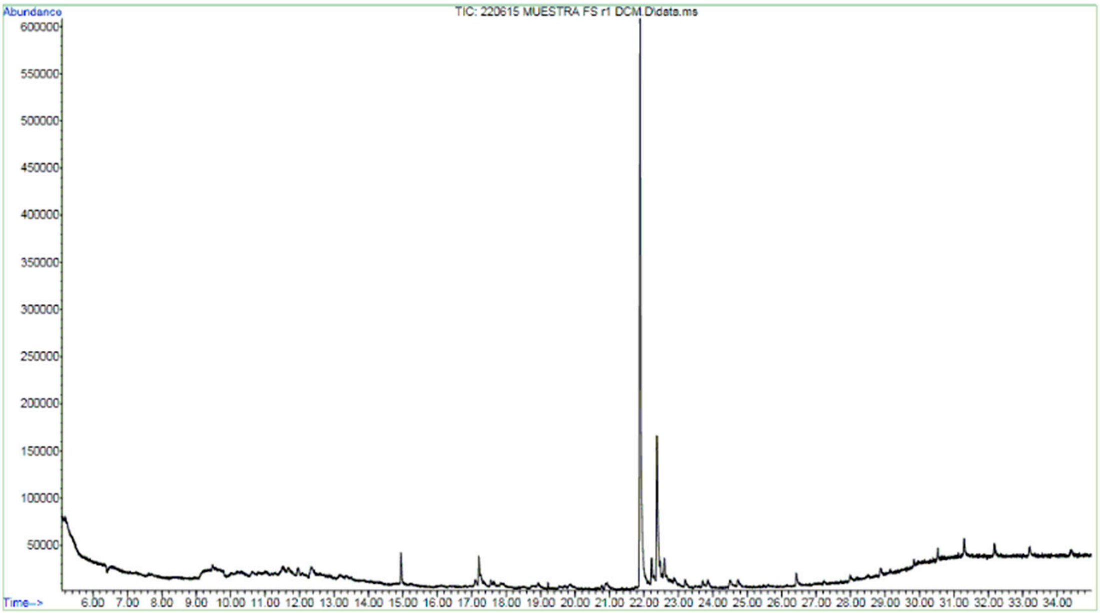

Figure S7 *- Chromatogram of sample FS - dichloromethane, obtained by CG-MS*

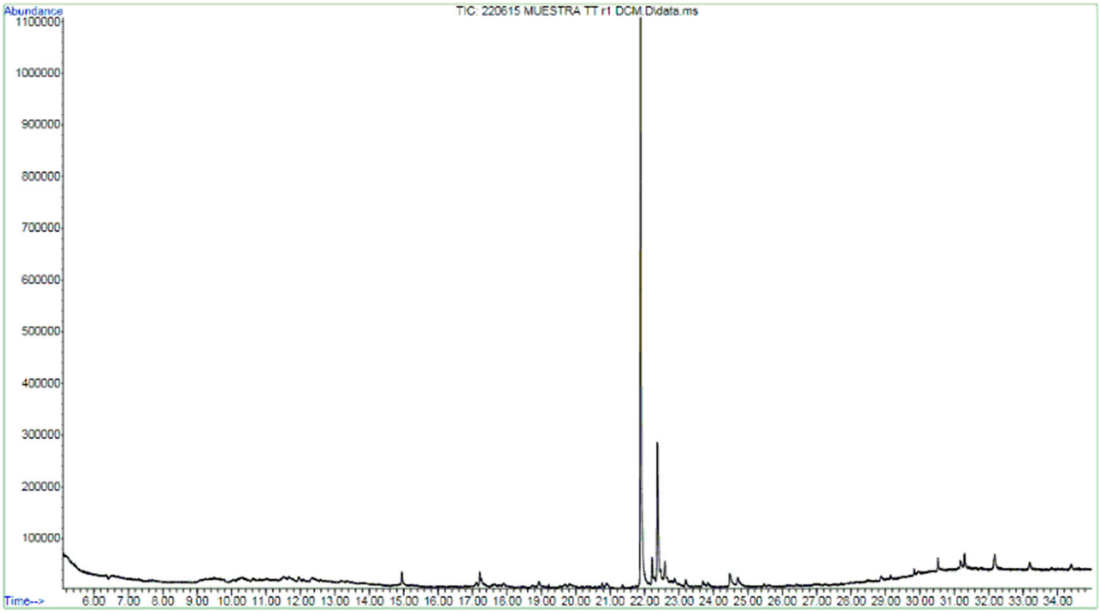

Figure S8 *- Chromatogram of sample TT - dichloromethane, obtained by CG-MS*

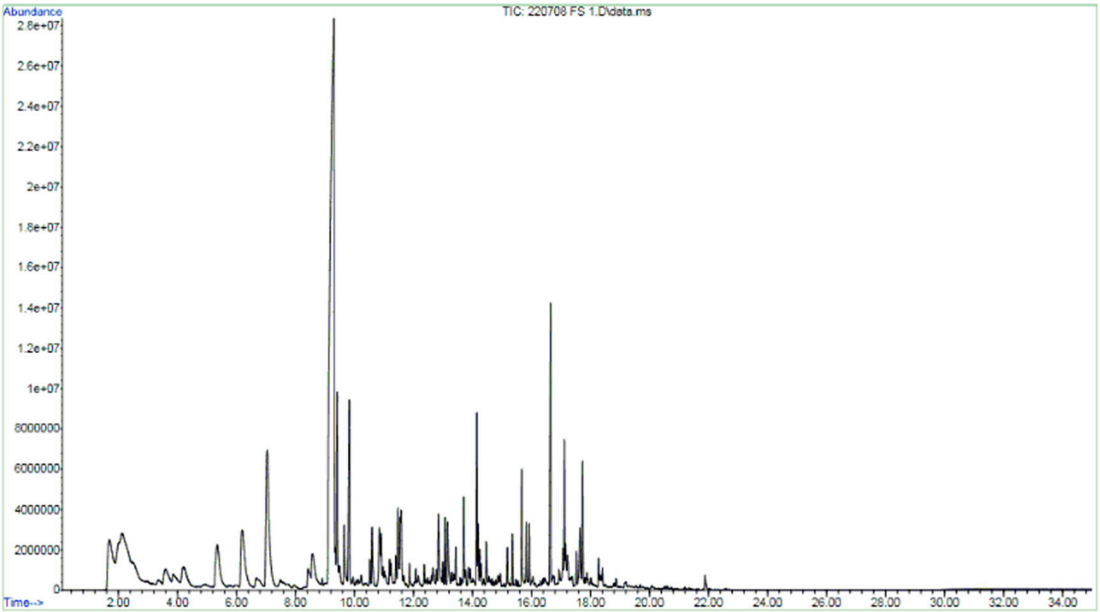

Figure S9 *- Chromatogram of sample FS (volatile compounds), obtained by HS-SPME-GC-MS.*

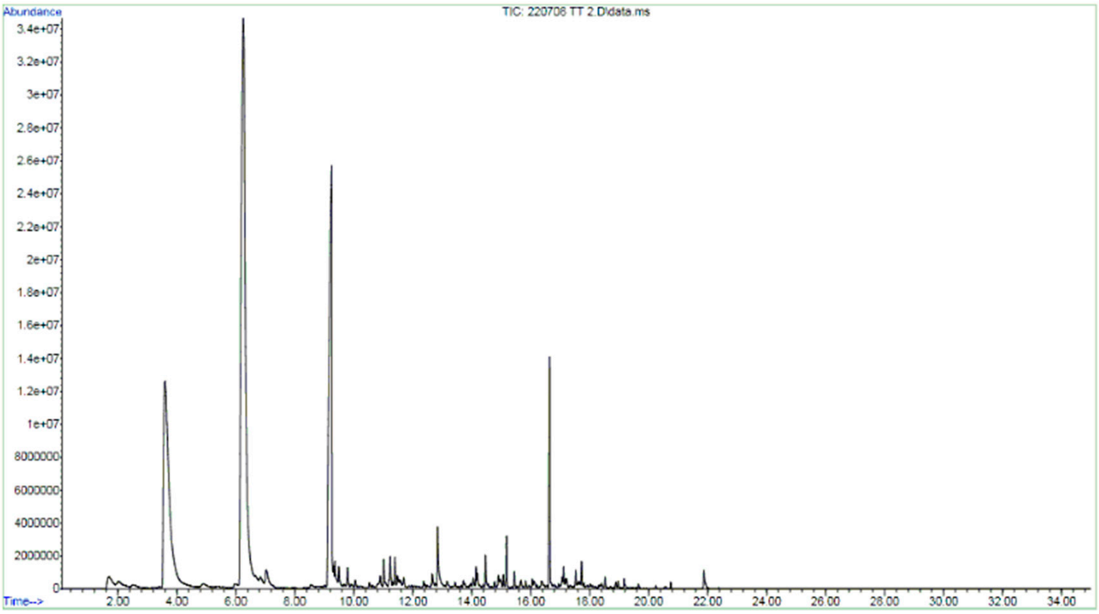

Figure S10 *- Chromatogram of sample TT (volatile compounds), obtained by HS-SPME-GC-MS.*

Table S7 shows the identification data of the non-volatile compounds obtained by UPLC-QTOF present in sample FS of the industrial annatto residue (replicas 1, 2 and 3) – positive mode

Table S7. Identification data of the non-volatile compounds obtained by UPLC-QTOF present in sample FS of the industrial annatto residue (replicas 1, 2 and 3)

**RT (min)**	**Molecular Formula**	**Adduct**	**$[M-H]^+$ Observed**	**$[M-H]^+$ Calculated**	**Similarity%**	**CAS (candidates)**	**Compounds Candidates**	**Fragments**
6.83	$C_{11}H_{16}O_2$	¬ + (+1H)	181,124	181,2550	79,57	15356-74-8	*Dihydroactinidiolide*	163,1131; 135,1178; 121,1023; 107,0869; 105,0715; 95,0870; 93,0709; 91,0556
7.94	$C_{15}H_{14}O$	¬ + (+1H)	211,1143	211,2840	87,03	1083-30-3	*Dihydrochalcone, 3-phenylpropiophenone*	
9.46	$C_{21}H_{31}O$	¬ + (+1H)	331,225	300,4860	8,72			
9.82	$C_{23}H_{36}O_4$	¬ + (+1H)	377,2667	377,5450	70,66			
10.02	$C_{23}H_{38}O_4$	¬ + (+1H)	379,2825	379,5610	59,78			
11,51	$C_{40}H_{60}O_3$	¬ + (+1H)	589,4591	589,9250	47,6			
11.88	$C_{41}H_{66}O_5$	¬ + (+1H)	639,4659	639,9820	38,29			
12.33	$C_{39}H_{66}O_5$	¬ + (+1H)	615,4968	615,9600	62			
12.40	$C_{39}H_{69}O_5$	¬ + (+1H)	641,5118	618,9840	29,16			
12,58	$C_{40}H_{66}O$	¬ + (+23Na)	585,5016	585,9480	66,4			No fragments
12.72	$C_{42}H_{83}NO_6$	¬ + (+1H)	698,629	699,1350	91,04		*2,3-Dihydroxy-N-[(2S,3S,4R,8E)-1,3,4-trihydroxy-8-octadecen-2-yl]tetracosanamide*	586,5037; 585,5006; 557,4462; 369,2934;286,2328
12.96	$C_{39}H_{68}O_5$	¬ + (+1H)	617,5123	617,9760	84,69	15818-46-9	*1,3-dilinolein*	321,2105
13,17	$C_{41}H_{70}O_5$	¬ + (+1H)	643,5281	644,0140	47,16			
13,24	$C_{40}H_{60}O_2$	¬ + (+1H)	573,4649	573,9260	59,81			

n=3

Table S8 shows the identification data of the non-volatile compounds obtained by UPLC-QTOF present in sample TT of the industrial annatto residue (replicas 1, 2 and 3) – positive mode

Table S8. Identification data of the non-volatile compounds obtained by UPLC-QTOF present in sample TT of the industrial annatto residue (replicas 1, 2 and 3)

RT (min)	Formula molecular	Adduct	$[M-H]^+$ Observed	$[M-H]^+$ Calculated	Similarity%	CAS (candidates)	Compouds Candidates	Fragments
8.13	$C_{18}H_{37}NO_3$	¬ + (+1H)	316,2862	316,5060	91.21		*Halisphingosine A*	298,2758; 280,2655;60,0461
8.35	$C_{19}H_{37}NO_3$	¬ + (+1H)	328,2855	328,5170	76.56	56255-31-3	*Palmitoyllanine; N-acyl-L-amino acid*	310,2747; 298,2747; 280,2643; 88,0768;74,0611
						2421-33-2	*Palmitoyl sarcosine*	310,2747; 298,2747; 281,2682; 280,2643
							N-tetradecanoyl- Valine	310,2747; 298,2747; 281,2682; 280,2643
							Ethyl N-acetyl-N-dodecyl-β-alaninate	310,2747; 298,2747
9.24	$C_{18}H_{34}O_3$	¬ + (+23Na)	321,2409	321,4570	99.18			sin fragmentos
9.31	$C_{31}H_{57}NO_9$	¬ + (+1H)	588,4109	588,8030	80.89			sin fragmentos
9.57	$C_{20}H_{32}O_3$	¬ + (+1H)	321,2432	321,4810	99.32	54845-95-3	*icomucret*	265,1811; 179,1438
9.95	$C_{40}H_{80}N0_3$	¬ + (+1H)	622,6138	623,0840	36.42			
10.04	$C_{20}H_{34}O_3$	¬ + (+1H)	323,2592	323,4970	87.58	41530-90-9	*2,6,16-Kauranetriol*	305,2485
10.44	$C_{36}H_{69}NO_3$	¬ + (+1H)	564,5359	564,9600	62.75	5966-28-9	*Ceramide (d18:1/9Z-18:1)*	284,2960; 73,0479
						5966-28-9	*N-oleoyl-D- sphingosine; (9E)-N-[(2S,4E)-1,3-Dihydroxy-4-octadecen-2-yl]-9-octadecenamid*	284,2960; 73,0479
							(9E,12E)-N-(1,3-Dihydroxy-2-octadecanyl)-9,12-octadecadienamide	284,2960; 73,0479
10.51	$C_{36}H_{66}O_5$	¬ + (+23Na)	601,4818	601,9090	38.83			
10.68	$C_{37}H_{12}N_6$	¬ + (+1H)	541,1221	541,5530	2.95			
11.51	$C_{20}H_{32}O_3$	¬ + (+1H)	321,243	321,4810	87.94	same compound identified with an RT of 9.57.		
						54845-95-3	*icomucret*	265,1813; 179,1442

						70608-72-9	*(5S,6E,8Z,11Z,14Z)-5-Hydroxy-6,8,11,14-eicosatetraenoic acid; 5-HETE*	265,1811
12.56	$C_{40}H_{64}$	¬ + (+1H)	545,5092	545,9600	81.08	540-04-5	*(all-E)-Phytoene; (E/Z)-Phytoene*	
						13920-14-4	*Phytoene; 15-cis-phytoene*	81,0709
								81,0709
12.59	$C_{42}H_{64}O$	¬ + (+1H)	585,5016	585,9810	66.28		*sin resultados en el ChemSpider®*	
13.22	$C_{40}H_{60}O2$	¬ + (+1H)	573,4656	573,9260	65.75			
							kitol	81,0713
13.86	C57H76NO	¬ + (+1H)	790,5927	791,2410	10.76			

n=3

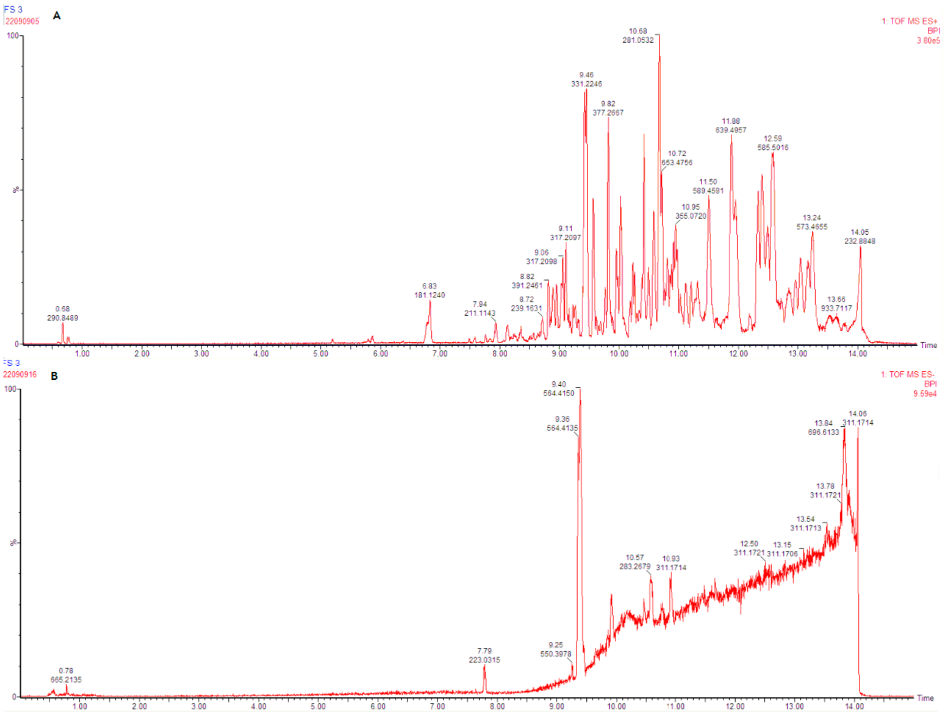

Figure S11 *- Chromatogram of sample FS (non-volatile compounds), obtained by UPLC-QTOF, (A - posity mode, B - negative mode).*

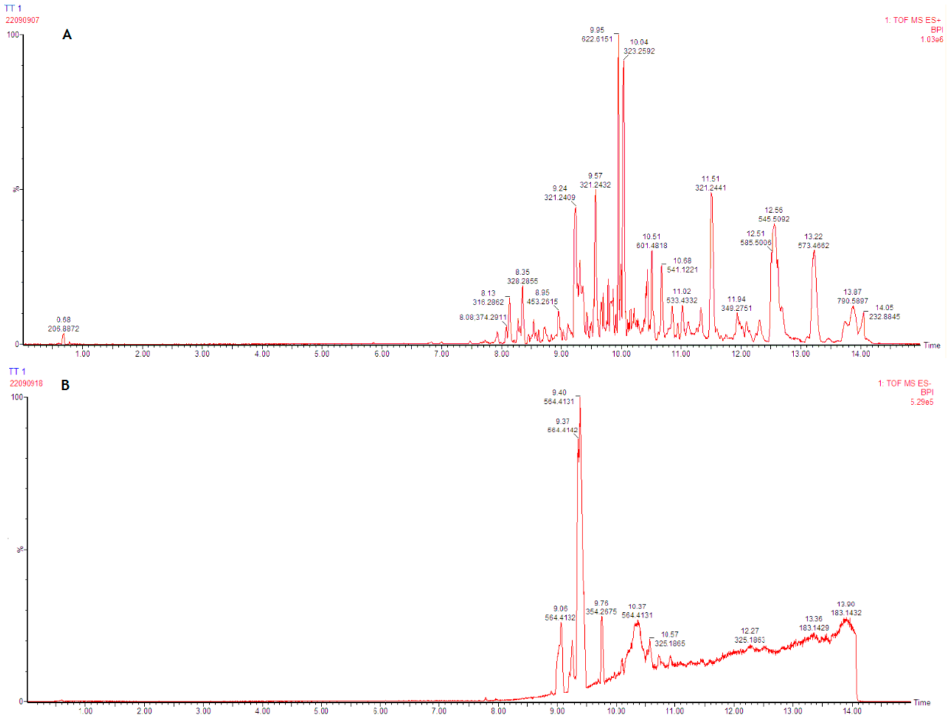

Figure S12 *- Chromatogram of sample TT (non-volatile compounds), obtained by UPLC-QTOF, (A - posity mode, B - negative mode).*
